# History of Dentistry on the Pacific Coast

**Published:** 1862-11

**Authors:** H. H. Black

**Affiliations:** Portland, Oregon


### Correspondence.

### HISTORY OF DENTISTRY ON THE PACIFIC COAST.

Editors Register :—In an effort to comply with your late request, in " writing an account of the state of the profession in this section of country," I feel that what I shall be able to communicate will be of little interest to the readers of the *Register*, inasmuch as such an account must necessarily contain much local matter, that is not strictly appropriate for the pages of a Dental Journal. If, however, your readers will pardon me for diverging somewhat from the subject-matter that should generally characterize a dental correspondence, I will endeavor to comply with your wishes, by making the account as brief and comprehensive as possible.

The first dental practitioner we have any knowledge of visiting the Pacific coast was Dr. Geo. Wellington, who accompanied the Hudson Bay mission to the Columbia River, where they founded Fort Van Couver, in 1824. Some eight years subsequently, Dr. Talliaferro, a French dentist of some note, visited Fort Van Couver and other points on the Columbia River. In 1836 Drs. Wellington and Talliaferro returned to Europe, thus depriving those in command of the forts of the services of a dentist. Dr. Talliaferro has since published in the *L' Echo Du Pacifique* a lengthy account of his practice and adventures in this region, which is deserving of much credit for its scientific information. In 1844 or 1846, Dr. Dunning, of New York, visited Astoria, at the mouth of the Columbia River. A few years later we learn of a Dr. Adams, of New Orleans, who it is claimed practiced in the coast towns of Mexico and Central America. In 1849, the discovery of rich gold mines in California gave the Pacific Coast a new impetus, and among the many that sought the new Eldorado was a proportionate number of dentists, the names of whom

I am unable to give; but believe Dr. Buckner, from Tennessee, among the first to practice dentistry in California. From that time to the present, the Pacific Coast has increased in population with a rapidity unparalleled in the history of the New World. The vast waste that but twelve years since expanded the entire North Pacific seaboard is now the home of a numerous and wealthy population, requiring the services of no less than three hundred dentists. Of this number, however, I regret to say at least one-half are not deserving the appellation they assume. But nevertheless we have some as good dentists as are in the United States, among whom it is due to mention Dr. G. Burdell, of San Francisco, who has done much to elevate the standard of the profession on the Pacific Coast.

Thus far all efforts at organizing an association for the mutual coöperation in enhancing the interests of the profession have failed, which is mainly attributable to the petty jealousies that characterize the mass of dentists in this country. Could we but exchange a few regiments of these "embryo tooth-carpenters" for a few of your Monumental, Quaker or Queen City dentists, it would not be our lot to witness as much ignorance and empiricism as at present. But it is quite evident we shall have to be content, for the present, without this exchange, as our country, in the opinion of *some* of our Cincinnati brethren, is "too remote for them." They are astonished that we should think to induce them to exchange a practice in Cincinnati, where they are receiving from $2 to $15 for a gold filling, and from $50 to $100 for a single set of teeth, for a practice in a "remote country," where they can only charge from $5 to $40 for a gold filling, and from $125 to $200 for a single set of teeth. From these facts, it would appear the country is "too remote," and we can not expect dentists in the East or South to locate in a country so "remote" that they can only charge three hundred per cent. more for their services.

In conclusion, I must briefly refer to the encouragement extended to dental literature by the profession on the Pacific Coast. Allow me to say it is truly surprising. Out of the three hundred practicing dentists that render the community *so much* service, there must be at least a *dozen* or *fifteen* that subscribe for a dental journal. In Oregon, however, many of our dentists are excusable, on the ground that they have yet to learn of such thing as a dental journal. But of those that have acquired this knowledge, they are either too indolent to read them, or *minus* the money to pay for them, and consequently the subscription list is entirely blank, with but one exception. It is due to mention, however, that the majority of these "Knights of the Excavator" receive the "Price Catalogues" from the depots regularly, and some of them are even in possession of the first edition of Harris' Principles and Practice of Dental Surgery. In a recent conversation with one of this class of dentists, if such they may be called, I took occasion to solicit his subscription for the *Register*, when I was made the startling proposition, that if I would loan him my journals regularly, after reading them myself, he would be greatly favored thereby, and would return them immediately after reading them. This is certainly evincing a strong desire to encourage dental journals, but in rather an indirect manner.

The subject of the present "state of the profession in this section of country" requires a more elaborate notice, which it will receive in my next. H. H. BLACK.

PORTLAND, OREGON, Sept. 24, 1862.

